# Why Hysterectomy Rate are Lower in India

**DOI:** 10.4103/0970-0218.42065

**Published:** 2008-07

**Authors:** Amarjeet Singh, Arvinder Kaur Arora

**Affiliations:** Department of Community Medicine, PGI, Chandigarh, India; 1 Department of Community Medicine, PGI, Chandigarh, India

## Introduction

Compared to a higher frequency of hysterectomy (HT; 10-20%) in other countries(1) a lower rate (4-6%) has been reported from India.(2–5) However, the reasons for this lower rate have not been adequately researched. The present study was therefore planned to estimate the rate of HT in the adult women of study area, and to ascertain the factors associated with decision making regarding HT.

## Materials and Methods

Three roadside villages (population 9786) in *Panchkula* district, *Haryana*, were purposively selected for the study. Every second house of these villages was surveyed to register married woman aged above 15 years to make a list of HT cases. They were individually interviewed by a social worker to collect information on socio-demographic data, reproductive health profile, reason for HT, treatment-seeking pattern, money spent, relief obtained, and response of husband and the family to the HT. Consent of the respondents was taken for the interview.

## Results

Of the enlisted 1000 subjects, 7% women had undergone HT [Table 1]. Prevalence of HT was significantly more in women aged above 35 years (*P* < 0.001). Excessive menstrual bleeding was the main indication for HT (52/70;74%). Uterine prolapse (10) and fibroid (3) were the other indications. Duration of symptoms was 1 year or more in 59 (84%) cases (>10 years in 10 cases). Three or more treatment agencies were consulted by 62 (89%) cases. First person to be consulted was traditional birth attendant, health worker/faith healer or local unqualified registered medical practitioner in 21 cases. Private qualified doctors were consulted by 17 cases. Government doctors were consulted by 29 cases. Two cases consulted *Ayurvedic* doctors first. In all except seven cases, HT was done after the age of 30 years. In 46 (66%) cases, the operation was done more than 1 month after it was advised (in 15 cases the lag was >12 months), in rest 24 (34%) cases, it was done within 1 month after it was advised. Reason for such gap was fear of operation, lack of money, ‘problem was tolerable’, ‘children were small’ etc.

**Table 1:** Agewise prevalence of hysterectomy in study women

Age (years)	Total women screened	Hysterectomy done	
		
		Number	%
15-24	118	-	-
25-34	293	8	2.7
35-44	241	21	8.7
45-54	165	25	15.1
≥55	183	16	8.7
Total	1000	70	7.0

x^2^–29.4, d.f - 2, *P* < 0.001

More than half of the women (41; 58.5%) told that they were afraid of the operation when they were first advised about HT. Majority of the husbands escorted the wives (55; 78.6%) and stayed with them in hospitals (48; 68.6%).

In almost half of the cases, HT was done in government hospitals (33; 47.1%). Four or more hospital visits were made before operation by the women in 45 (64%) cases. After operation, three or more follow-up hospital visits were made in 25 (35.6%) cases. Hospital stay was for less than 7 days in 16 (22.8%) cases, 8-15 days in 51 (72.8%) cases and for more than 15 days in three cases. In 22 (31.4%) cases, Rs.5000-10,000/- were spent, and in 35 cases (50%), more than Rs.10,000/- was spent on HT.

No complication was reported in 38 (54.2%) cases. In others, bleeding (5), fever (9), pain (4) or other problems were reported (incontinence, gas, backache, cough). Total relief was obtained after the operation in 43 cases (61.4%). In remaining 27 (38.5%) cases, some relief was reported. Some of the women reported late medical problems after HT, viz., backache (44; 62.9%) vaginal discharge (3; 4.3%), weakness (11; 15.7%), pain (11; 15.7%), weight gain (3; 4.3%), gas (7; 10%), incontinence (8; 11.4%) and difficulty in sitting/walking (5; 7.1%). In 10 (14.3%) cases, feeling of a ‘sense of incompleteness’ was reported after removal of uterus. Half of the women told that they had consulted a successfully operated case before deciding about HT. Only three (4.3%) women told that they repented their decision for operation, five (7.1%) said it was too early, three (4.3%) said it was avoidable and 11 (15.7%) said it was not necessary. Only one woman (1.4%) reported hormone replacement therapy (HRT) prescription after HT.

Eighteen (25.7%) women said that after HT they were relieved off the botheration about menses. In seven (10%) cases, sex life was affected. Visiting religious places (23; 33%), walking (13; 18.5%), washing clothes (15; 21.4%), mopping (11; 15.7%) and kitchen work (9; 12.8%) were also improved after operation. Sixteen (37.2%) respondents did not report any symptom after surgery, whereas 17 (39.5%) respondents complained of a sense of emptiness and six (14.0%) reported pain/fever and urinary problems.

## Discussion

Our earlier studies on uterine prolapse and menopause(1–6) had revealed that, for women in India, family responsibilities take precedence over their own health concerns. Even if they have any uterus-related disease, a desire to complete the desired family size often delays the decision to get one's uterus removed. So, in India, more often than not, the HT, even if indicated and advised, is delayed till the children are old enough and women have a sense of having fulfilled their family commitments. Our study revealed that prevalence of HT rose with age, maximum being in 45-54 year age group.

Routine life was affected after HT in 11-25% respondents of our study. Though in terms of money spent, the number of visits made to the hospitals and duration of hospitals stay, HT appeared to be a major drag on family resources, yet, it provided relief from the symptoms in all the cases. Uterus as the childbearing organ with regular monthly menstrual bleeding is usually identified as an essence of womanhood in India. It was natural that, after HT, many of our respondents felt that they were not a woman anymore. Similar feelings have been reported by menopausal women in our earlier studies.(1–6) As per biomedical perspective, physically uterus occupies a very small space in abdomen. So, its removal should not be physically felt by the women. However, it seems that the lay women's conceptual framework of reproductive health assigns a large space for uterus in abdomen so much so that after HT they reported a sense of emptiness inside. Some even complained that, after HT, they were not able to tie their *pajama* cord since nothing was left in the abdomen to support the knot.

In the West, gynecologists have been criticized in general because it is alleged that they perform HT rather too readily. In fact, the high incidence of HT in the West was highlighted as the manifestation of misuse of gynecological surgery to control women.(1) Lower rate for HT in the study (7%) as compared to West (10-20%) and the longer treatment lag observed in our study may be because of various reasons, viz., considerably lower level of medicalization of menopause among women, their lower status in society, poverty, illiteracy ‘culture of silence’ fear of operation and the fact that their tolerance threshold was higher and that they considered menopausal symptoms as a part of life and had a positive view of menopause.(1–5)

In Western countries, uterine fibroid and bleeding are the common indications. Present study also revealed that uncontrolled bleeding was the most common reason for HT (39%). Surprisingly, in our study, HRT was prescribed in one case only, whereas in western countries and in urban India, more of HT cases are prescribed HRT.(1–6)
